# Dynamic learning of the meaning of information changes pain perception

**DOI:** 10.1038/s41598-025-14299-z

**Published:** 2025-10-14

**Authors:** Emily J Hird, Christiana Charalambous, Wael El-Deredy, Anthony K. P. Jones, Deborah Talmi

**Affiliations:** 1 Institute of Cognitive Neuroscience, University College London, Alexandra House, 17 Queen Square, London, UK; 2 Department of Mathematics, University of Manchester, Manchester, UK; 3 Centro de Investigación y Desarrollo en Ingeniería en Salud, Universidad de Valparaiso, Valparaiso, Chile; 4 Division of Neuroscience and Experimental Psychology, University of Manchester, Manchester, UK; 5 Human Pain Research Group, Salford Royal NHS Foundation Trust, Manchester, UK; 6 Department of Psychology, University of Cambridge, Downing Site, Cambridge, UK

**Keywords:** Pain, Placebo analgesia, Expectation, Bayesian reinforcement learning, Psychology, Learning and memory, Sensory processing

## Abstract

Beliefs influence the intensity of perceived pain. We previously applied regression models to two independent pain-cueing datasets (https://osf.io/5r6z7/) and found that pain intensity reports were a function of the error between participants’ beliefs and the actual stimulation they received, such that greater error decreased the influence of prior beliefs. Although this result appeared to present a challenge to established models of perception, our former analyses did not formally compare the result against established computational models. Here (https://osf.io/fj27k/) we compared a model that corresponded to our original interpretation against Bayesian reinforcement learning models. We found that pain intensity perception was best explained by a model in which the expected value of each information cue was updated via a Bayesian reinforcement learning algorithm. In addition to their importance for understanding the fundamental mechanism that underlies pain perception, these new results indicate that deception pain studies should not assume that participants’ belief about the information they are given is fixed. Rather, our results provide evidence that the meaning of cues change dynamically over the course of the session through trial-by-trial updates, even when participants are instructed otherwise.

**Supplementary Information:**

The online version contains supplementary material available at 10.1038/s41598-025-14299-z.

## Introduction

Beliefs influence pain perception, such that pain reports are a function of the error between what participants believe and the stimulus intensity. These observations, from clinical and laboratory experiments, have supported predictive processing^1^ and reinforcement learning^2^ accounts of pain perception. According to predictive processing theory, the brain makes Bayesian inferences about the causes of sensory inputs by weighting them against prior beliefs^3–7^. This framework has been argued to account for pain perception^1,8^. It explains why pain perception is biased towards beliefs^3,9–12^ and why more precise beliefs influence pain perception more^4,13–16^. Recent computational modelling work has supported this framework (reviewed in:^17^. While the priors within predictive processing models are typically based on the mean and variance of fixed cues, in Bayesian reinforcement learning models, these values are updated in each trial based on the error between a prediction and the actual outcome^18,19^. This framework has been successfully applied to pain intensity ratings, suggesting that people update and integrate their beliefs about pain into pain perception^20–22^.

We previously showed that on any given pain experience, with greater error, the influence of belief on pain perception decreases when the discrepancy between belief and stimulus intensity (i.e., prediction error) is large^23^. We interpreted this as evidence for an immediate, error-sensitive scaling of belief influence—a functional mechanism not explicitly considered in standard predictive processing models. Specifically, we proposed that when prediction errors exceed an individually set threshold, the influence of beliefs on perception diminishes. These results, replicated across two datasets, appeared to challenge predictive processing theory by identifying a dynamic, within-trial modulation of belief impact based on prediction error magnitude. However, our interpretation could not be verified without formally comparing our model to predictive processing models, including Bayesian reinforcement learning models. Furthermore, although we assumed that cued beliefs were fixed over the course of the experiment, we observed that the effect of cues changed across experimental trials, hinting towards time-dependent changes in the influence of cues on pain perception, despite the cues having a fixed nominal value and our instructions to participants that the cue was always truthful. These time-dependent effects suggested that participants re-evaluated the cues over the session, which our models did not consider. It is feasible that up to a certain threshold, cue-elicited beliefs are fixed, but after encountering a certain size of prediction error, participants become more dynamic in their belief updating as they expect the environment to become less reliable. In the current study, we compared the fit between established models of pain perception and our published model. This analysis is important because it compares basic predictive processing models and those that consider changes in beliefs inspired by cues over time, and because to date, modelling of deceptive cued pain tasks have seldom considered changes to the value of the cue within experimental sessions.

We assessed the fit of a Bayesian model where beliefs that are more discrepant to the painful stimulus have less influence on pain perception, as in our original interpretation^23^ (*Model 1*), and which does not consider trial-by-trial learning; and a Bayesian model where cues become less credible over the course of the experiment as participants encounter discrepant stimuli (*Model 2*). We also considered a Bayesian reinforcement learning model in which beliefs are updated on each trial based on the prediction error (*Model 3*). We illustrate these 3 models in Fig. 2. We allowed each model to vary by individual because the importance of psychological influences on pain perception varies between individuals^24–26^.

## Materials and methods

### Participants

For both Dataset 1 and 2, we recruited participants via university advertisements, for which they received £15 compensation. Participants had normal or corrected-to-normal vision, no history of neurological or psychiatric conditions, had not taken analgesics on the day of the experiment, and did not have a history of chronic pain. Ethical approval was granted by the University of Manchester, where the study took place. All methods were carried out in accordance with the Code of Ethics of the World Medical Association (Declaration of Helsinki). Informed consent was obtained from all subjects.

For Dataset 1, 31 participants aged 18–35 (19 females, mean age 23 years), and for Dataset 2, 30 participants (15 females, mean age 21 years) were recruited into the study. A sample size of *N* = 30 is sufficient to have power of 0.8 to detect a medium to large effect sizes in within-participant comparisons, such as the difference between their responses to the same stimulus under different expectation conditions, assuming alpha of 0.05. This resembles the sample size in previous studies investigating the effect of expectation or uncertainty on electrical pain intensity rating, which typically recruit 15–30 participants^4,10,27^. The study for Dataset 1 and Dataset 2 only differed in terms of the experimenter collecting the data, and the room the data were collected in, which were nonetheless similar in shape, size and light level. They took place approximately 9 months apart.

### Apparatus

Painful stimuli were electrical pulses (pulse width: 5 milliseconds) delivered via a concentric electrode by a constant current stimulator (Digitimer DS5 2000, Digitimer Ltd., Welwyn Garden City, UK). All stimuli were controlled through a Matlab platform (Mathworks) which interfaced with the pain stimulator via a digital-to-analogue convertor (Multifunction I/O device, National instruments, Measurement House, Berkshire, UK). Participants submitted their intensity ratings of the pain using a keypad. Visual cues were presented on a desktop computer screen one metre away from the participant.

### Procedure

This section has been taken directly from our previous paper^23^ which reports collection of the data modelled here. The experimenter introduced the study as a test of pain perception. After participants consented, the concentric electrode was attached to the back of the participant’s hand. Participants underwent a pain calibration staircase procedure on their left hand to determine their response to increasing electrical stimulus intensities. The stimulus intensity increased to a maximum of five volts. We used a 0–10 Numerical Pain Scale (NPS) to measure the pain intensity rating, where a pain intensity rating of NPS 2 was when the stimulus became “just painful”, NPS 5 was “medium pain”, and NPS 8 was at the point where stimulus was “just tolerable”, replicating previous research^10,28^. We repeated this procedure three times and computed the average stimulus intensities over these three repetitions corresponding to NPSs 2, 3, 4, 5, 6, 7 and 8. Participants then underwent a test procedure: stimulus intensities corresponding to their pain intensity ratings NPS 2 to 8 were delivered in a pseudo-randomised order four times and participants were instructed to identify the intensity of each pulse. Participants had to correctly identify 75% of stimulus intensities to continue to the main experiment. If they did not achieve this in the test procedure, the intensities were adjusted (intensity was increased if participants rated the stimulus intensity as lower than in the pain calibration procedure, and vice versa), and the test repeated until participants correctly identified 75% of stimulus intensities.

**Fig. 1:**
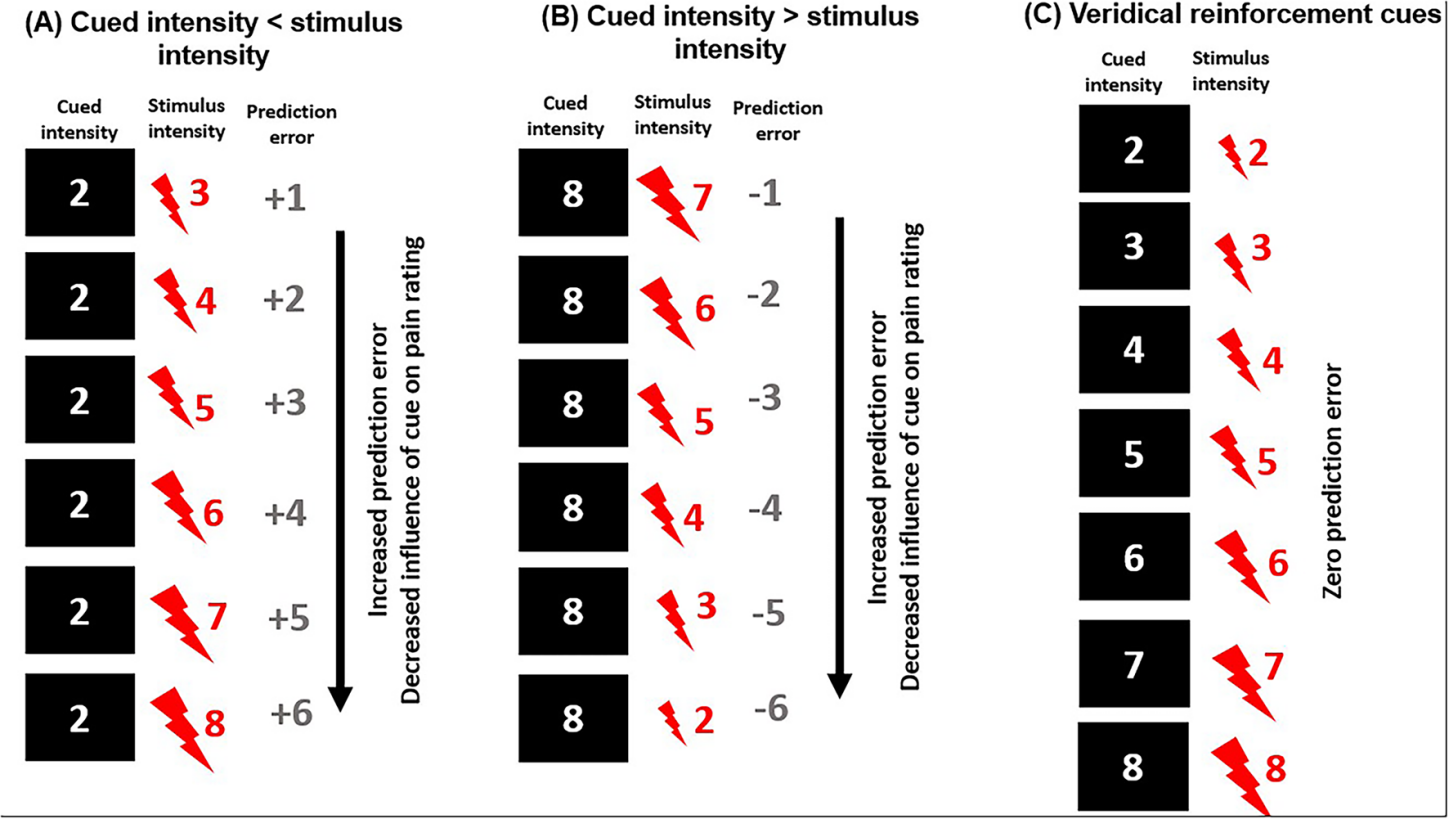
Participants viewed a number on a computer screen from 2 to 8 which cued intensity for that Trial. Importantly, participants were instructed that the cue was always truthful. Participants received the stimulus, then a numerical pain scale was presented asking participants to rate the stimulus intensity. Erroneous cues were followed by a stimulus intensity that was higher (A) or lower (B) than the cued intensity, to generate varying degrees of prediction error. The results of our previous analysis^23^ suggested that past an individually-set boundary, increased prediction error was associated with a decreased influence of cued intensity on pain rating. We also included veridical cues, followed by a stimulus intensity that matched the cued intensity, to maintain participant’s belief in the cues (right), which was associated with zero prediction error.

In the main experiment, participants were instructed that the cue predicted the stimulus intensity on each trial. The cue was a number on the computer screen which depicted the intensity of the upcoming stimulus (Fig. 1), and then a stimulus intensity was delivered in a partially reinforced cueing procedure. The number of trials in each condition is detailed in Table 1. Whilst half of the NPS 2 (“just painful”) and NPS 8 (“highest tolerable pain”) cues were followed by unexpected stimulus intensity, all other pain cues were veridical, which served to reinforce participant’s belief in the validity of the cues. Participants were instructed to rate the intensity of the stimulus and were not informed that the cues were discrepant. Trials were randomised across participants. On each experimental trial, participants viewed a fixation cross, a cue, and then a blank screen. The stimulus was delivered, and a screen was presented which prompted participants to numerically rate their perceived pain intensity on a 0–10 NPS using a keypad. There was no time limit on this response.

**Table 1:** A summary of all Trials.

Cued intensity	Stimulus intensity	PE	Number of trials
2	2	0	5
2	3	1	5
2	4	2	5
2	5	3	5
2	6	4	5
2	7	5	5
2	8	6	5
3	3	0	10
4	4	0	10
5	5	0	10
6	6	0	10
7	7	0	10
8	8	0	5
8	7	-1	5
8	6	-2	5
8	5	-3	5
8	4	-4	5
8	3	-5	5
8	2	-6	5

Cued intensity: the level of pain stimulus intensity communicated by the visual numerical cue prior to the painful stimulus; stimulus intensity: the true intensity of the stimulus, as rated in the pain calibration procedure; PE: prediction error (stimulus intensity – cued intensity); number of trials: number of trials for that condition in the experimental session.

In Dataset 1, an error in the experimental script meant that each subject received one extra trial from the condition ‘Cued intensity 2 Stimulus intensity 4’ and one fewer trial from another random condition. Dataset 2 was not affected by this issue. All data and analysis code are available at https://osf.io/fj27k/.

### Modelling

We consider several models, all of which inherently have the same underlying structure, to describe the distribution of the pain rating, after considering the effect of cue and stimulus, as well any prior traits/beliefs, such as optimism/pessimism. Table S1 in the Supplementary Materials provides an explanation of each parameter across the three models.

We propose the following density for the reported pain rating of participant $\:i$ in trial$\:\:j$, $\:{R}_{ij}\:$:

1 $$\:f\left({R}_{ij}\right|{X}_{ij},\:{Z}_{ij})\:\propto\:{f}_{1}\left({Z}_{ij}\right|{R}_{ij},\:{X}_{ij})\:{f}_{2}\left({X}_{ij}\right|{R}_{ij}\left)\:{f}_{3}\right({R}_{ij})$$

where $\:{X}_{ij}$ is the stimulus, $\:{Z}_{ij}$ is the cue and $\:i\:=\:1,\dots\:,n;\:j\:=\:1,\dots\:,{m}_{i}$, with $\:n$ being the number of participants and $\:{m}_{i}\:$the total number of trials for participant $\:i$. In other words, we assume that up to proportionality, the density of the pain rating can be decomposed into three parts: the first component, $\:{f}_{1}\left({Z}_{ij}\right|{R}_{ij},{X}_{ij})$, describes the effect of the cue conditional on the stimulus, the second component, $\:{f}_{2}\left({X}_{ij}\right|{R}_{ij})$, contains the effect of the stimulus independent of the cue and finally, the last component, $\:{f}_{3}\left({R}_{ij}\right)$, accounts for any trait like effects. Under all models, we assume that $\:{f}_{1}$, $\:{f}_{2}$ and $\:{f}_{3}$ are Normal densities, implying that $\:f\:$will be proportional to a Normal density. Further distributional assumptions regarding the way the cue distribution is modulated result in different models.

#### PE-modulated bayesian model (Model 1)

$$\:{{Z}_{ij}|R}_{ij},\:{X}_{ij}\:\sim\:N({R}_{ij},\:{\rho\:}_{PE}^{2})$$

$$\:{X}_{ij}|{R}_{ij}\:\sim\:N({R}_{ij},\:{\beta\:}_{i}^{2})\:$$

2 $$\:{R}_{ij}\:\sim\:N({\mu\:}_{i},\:{\nu\:}_{i}^{2})$$

In the *PE*-modulated Bayesian model (Model 1) beliefs that are more discrepant to the painful stimulus have less influence on pain perception. This is achieved by assuming the variance in the cue Normal distribution, $\:{\rho\:}_{PE}^{2}$, is a function of the prediction error, $\:PE\:=\:X\:-\:Z$. In other words, $\:{\rho\:}_{PE}^{2}={\rho\:}^{2}g\left(\left|PE\right|\right)$, where $\:g$ is a function to be determined. In the supplementary materials we explain why we consider these three forms for $\:g$ (S1. Modelling, and Figure S1). These considerations led us to develop 3 variants of the PE-modulated model:

- Model 1.1: $$\:{\rho\:}_{PE}^{2}\:=\:{\rho\:}_{i}^{2}\left|P{E}_{ij}\right|$$
- Model 1.2: $$\:{\rho\:}_{PE}^{2}\:=\:{0.2\rho\:}_{i}^{2}\text{e}\text{x}\text{p}\:|P{E}_{ij}/2|$$
- Model 1.3: $$\:{\rho\:}_{PE}^{2}\:=\:{\rho\:}_{i}^{2}\:\text{l}\text{o}\text{g}\:\left|P{E}_{ij}\right|$$

Note that the formulation above for $\:{\rho\:}_{PE}^{2}$ automatically allows the cue variance to vary with trial, because it depends on $\:PE$, which changes for each participant in each trial.

#### Time-modulated bayesian model (Model 2)

$$\:{{Z}_{ij}|R}_{ij},\:{X}_{ij}\:\sim\:N({R}_{ij},\:{\rho\:}_{Trial}^{2})$$

$$\:{X}_{ij}|{R}_{ij}\:\sim\:N({R}_{ij},\:{\beta\:}_{i}^{2})\:$$

3 $$\:{R}_{ij}\:\sim\:N({\mu\:}_{i},\:{\nu\:}_{i}^{2})$$

In the time-modulated Bayesian model (Model 2), cues become less credible gradually, over the course of the experiment as participants encounter discrepant stimuli. This gradual, non-specific linear modulation is simulated by assuming that the variance in the cue distribution, $\:{\rho\:}_{Trial}^{2}$, is a linear function of the Trial number. In other words, Model 2 assumes that $\:{\rho\:}_{Trial}^{2}\:=\:{\rho\:}_{i}^{2}j\:$where $\:j\:=\:1,\dots\:,{m}_{i}$.

#### Bayesian reinforcement learning model (Model 3)

In this Bayesian RL model (Model 3), beliefs are updated on each trial based on the experience each participant had with the same cue. Participants update the pain intensity they expect when the nominal cue is 2 every time they are presented with that cue, and similarly when the nominal cue is 8. We consider two Bayesian RL models, each of which updates the cue distribution in a different way; we denote these as the implicit and explicit RL models. Both assume the following density for $\:{R}_{ij}:$

4 $$\:f\left({R}_{ij}\right|{X}_{ij{\prime\:}},\:{Z}_{ij}^{*},{Z}_{ij-1}^{*})\:\propto\:{f}_{1}\left({Z}_{ij}^{*}\right|{R}_{ij},\:{X}_{ij{\prime\:}},{Z}_{ij-1}^{*})\:{f}_{2}\left({X}_{ij}\right|{R}_{ij}\left)\:{f}_{3}\right({R}_{ij})$$

where $\:{X}_{ij{\prime\:}}={\stackrel{-}{X}}_{ij}$ for the implicit RL model and $\:{X}_{ij{\prime\:}}={X}_{ij-1}$ for the explicit RL model. Note that the density ( 4 ) takes the same form as in ( 1 ), with the cue, $\:{Z}_{ij}$ replaced by the expected cue, $\:{Z}_{ij}^{*}$. Distributions for the expected cue, stimulus and prior expectations remain Normal:

$$\:{{Z}_{ij}^{*}|R}_{ij},\:{X}_{ij{\prime\:},}\:{Z}_{ij-1}^{*}\:\sim\:N({R}_{ij},\:{\rho\:}_{i}^{2})$$

$$\:{X}_{ij}|{R}_{ij}\:\sim\:N({R}_{ij},\:{\beta\:}_{i}^{2})\:$$

5 $$\:{R}_{ij}\:\sim\:N({\mu\:}_{i},\:{\nu\:}_{i}^{2})$$

In the implicit RL model, denoted Model 3.1, the $\:{Z}_{ij}^{*}$ are the expected values of the cue obtained via a Bayesian updating procedure, as follows:

$$\:\:{Z}_{ij}^{*}={\sigma\:}_{ij}^{2}\left(\frac{{Z}_{ij-1}^{*}}{{\sigma\:}_{ij-1}^{2}}+\frac{{n}_{j}{\stackrel{-}{X}}_{ij}}{{S}_{ij}^{2}}\right)$$

where $\:{\sigma\:}_{ij}^{2}=\:{\left(\frac{1}{{\sigma\:}_{ij-1}^{2}}+\frac{{n}_{j}}{{S}_{ij}^{2}}\right)}^{-1}$, $\:{\stackrel{-}{X}}_{ij}=\frac{1}{{n}_{j}}\sum\:_{k=1}^{j}{X}_{ik}\:$, $\:{S}_{ij}^{2}=\frac{1}{{n}_{j}-1}{\sum\:_{k=1}^{j}\left({X}_{ik}-{\stackrel{-}{X}}_{ij}\right)}^{2}$and $\:{n}_{j}\:$is the length of $\:{(X}_{i1},\dots\:,\:{X}_{ij})$. Also, $\:{Z}_{ij}^{*}$ is initialized either at 2 or 8 and $\:{\sigma\:}_{i1}^{2}=0.1.\:$Based on this model, we assume that the cue is updated, trial-by-trial, considering both the history of cue values (through $\:{Z}_{ij}^{*}$ and $\:{\sigma\:}_{ij}^{2}$) and stimulus values (through $\:{\stackrel{-}{X}}_{ij}$ and $\:{S}_{ij}^{2}$). Though this is a learning model, its formulation prohibits us from capturing individual learning rates.

In the explicit RL model, denoted Model 3.2, we consider a varying learning rate when updating the expected cue, $\:{Z}_{ij}^{\text{*}}$. In particular, we assume

6 $$\:{Z}_{ij\:}^{*}={Z}_{ij-1}^{*}+\:{\alpha\:}_{i}\left({X}_{ij-1}-{Z}_{ij-1}^{*}\right)={Z}_{ij-1}^{*}+\:{\alpha\:}_{i}P{E}_{ij-1}^{*}\:\:$$

where $\:P{E}_{ij-1}^{\text{*}}$ is the prediction error based on the expected cue $\:{Z}_{ij-1}^{\text{*}}$, and $\:{\alpha\:}_{i}$ is the learning rate for participant $\:i$, which ranges between 0 (equivalent to $\:{Z}_{ij}^{\text{*}}={Z}_{ij-1}^{\text{*}}$, i.e. no learning) and 1 (equivalent to $\:{Z}_{ij}^{\text{*}}={X}_{ij-1}$, i.e. complete disregard for the cue). Note that in this model, $\:{Z}_{ij}^{*}$ is updated dynamically with $\:{\alpha\:}_{i}$, as opposed to Model 3.1, where $\:{Z}_{ij}^{*}$ is only computed once and remains static through the estimation process.

#### Estimation procedure

For estimation, we employ a Bayesian approach via Markov Chain Monte Carlo. For any of the models introduced so far, we can obtain the posterior distribution of the parameters $\:\theta\:$, where $\:\theta\:\:=\:({\beta\:}^{2},{\rho\:}^{2},\mu\:,{\nu\:}^{2})$ for Models 1, 2, and 3.1 and $\:\theta\:\:=\:({\beta\:}^{2},{\rho\:}^{2},\mu\:,{\nu\:}^{2},\:\alpha\:)$ for Model 3.2, using Bayes theorem:

$$\:\pi\:\left(\theta\:\right|R,X,Z)\propto\:f\left(R\right|X,\:Z,\theta\:)\:\pi\:\left(\theta\:\right)$$

$\:f\left(R\right|X,Z,\theta\:)\:=\:f(R|X,Z)$ as defined in ( 1 ) for Models 1 and 2 or ( 4 ) for Model 3 and $\:{\beta\:}^{2}=\left({\beta\:}_{1}^{2},\:\dots\:,\:{\beta\:}_{n}^{2}\right),\:\mu\:=\left({\mu\:}_{1},\:\dots\:,\:{\mu\:}_{n}\right),{\nu\:}^{2}=\left({\nu\:}_{1}^{2},\:\dots\:,\:{\nu\:}_{n}^{2}\right),\:\alpha\:=\left({\alpha\:}_{1},\:\dots\:,\:{\alpha\:}_{n}\right)$ and $\:{\rho\:}^{2}=\left({\rho\:}_{1}^{2},\:\dots\:,\:{\rho\:}_{n}^{2}\right)$. Also, $\:\pi\:\left(\theta\:\right)\:$is the prior distribution for the parameter vector $\:\theta\:$ and assuming a-priori independence between parameters, $\:\pi\:\left(\theta\:\right)\:=\:\prod\:_{\text{l}=1}^{\text{L}}\pi\:\left({\theta\:}_{\text{l}}\right)\:$, where $\:L\:=\:4n$ for Models 1, 2, and 3.1 and $\:L\:=\:5n$ for Model 3.2. In our Bayesian approach, we can sample each component in $\:{\beta\:}^{2},\:{\rho\:}^{2},\:\mu\:$ and $\:{\nu\:}^{2}$ directly from their conditional posterior distributions through a Gibbs sampling scheme; however, the components in $\:\alpha\:$ do not have a closed form posterior. Thus, we have implemented a Hamiltonian Monte Carlo algorithm for sampling the parameters in Model 3.2. Finally, for model comparison, we use the Deviance Information Criterion (DIC)^29^where in practice, a difference of more than 10 units is considered significant.

### Results

**Fig. 2:**
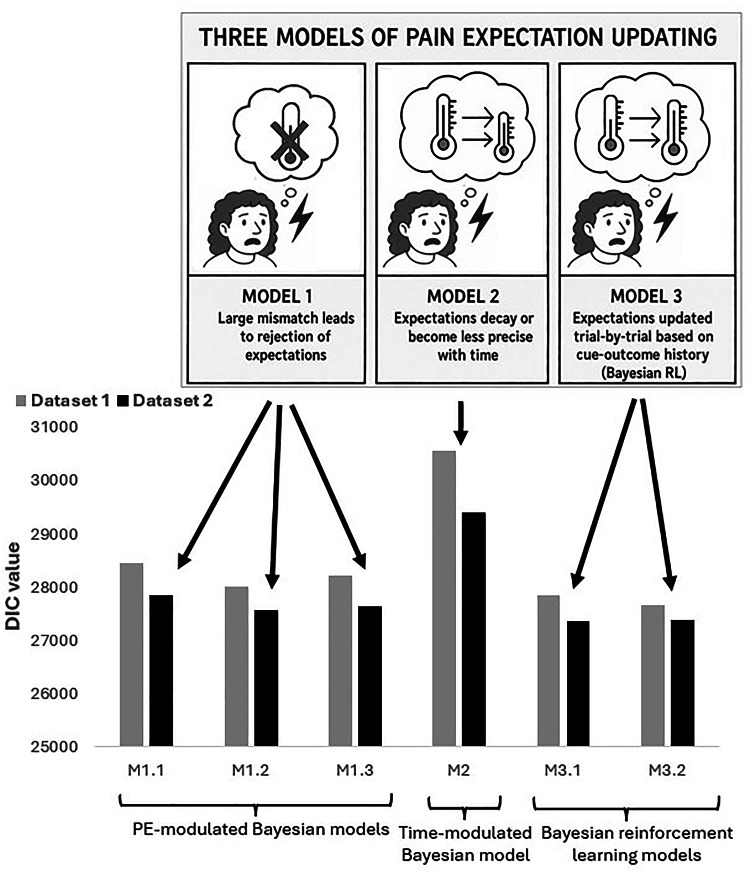
Illustration of the 3 models considered in this work. In the PE-modulated Model 1, we assume participants lose faith in the cue as the prediction error increases, where as in the Trial-modulated Model 2, this loss in faith is increases with time. Finally, in the Reinforcement Learning Model 3, participants can learn by dynamically updating their beliefs about the cue. DIC values for each model are depicted fitted to both Datasets. Models 3 outperform Models 1 and 2, indicated by their lower DIC values.

The results from fitting the models are summarised in Fig. 2 and in Supplementary Table S2. Out of Models 1 and 2, M1.2 (which assumed $\:{\rho\:}_{PE}^{2}\:=\:{0.2\rho\:}_{i}^{2}exp\:|P{E}_{ij}/2|$) was the best model across datasets. This model is closest to our published model^23^in terms of the belief that discrepant cues have less effect after a certain threshold. However, according to Fig. 2, the RL models outperformed this model. In particular, the explicit RL model which allowed a varying learning rate when updating the cue information (Model 3.2) fitted dataset 1 better, but for dataset 2, the implicit RL model (Model 3.1) was best. Although there was no agreement as to which RL model was the best overall, the differences in DIC between Models 3.1 and 3.2 for dataset 2 were small, such that we would consider Model 3.2 suitable for both datasets. The posterior means and 95% credible intervals for the parameters from the best model (3.2) are summarised in Fig. 4 for dataset 1 and Figure S2 for dataset 2.

**Fig. 3:**
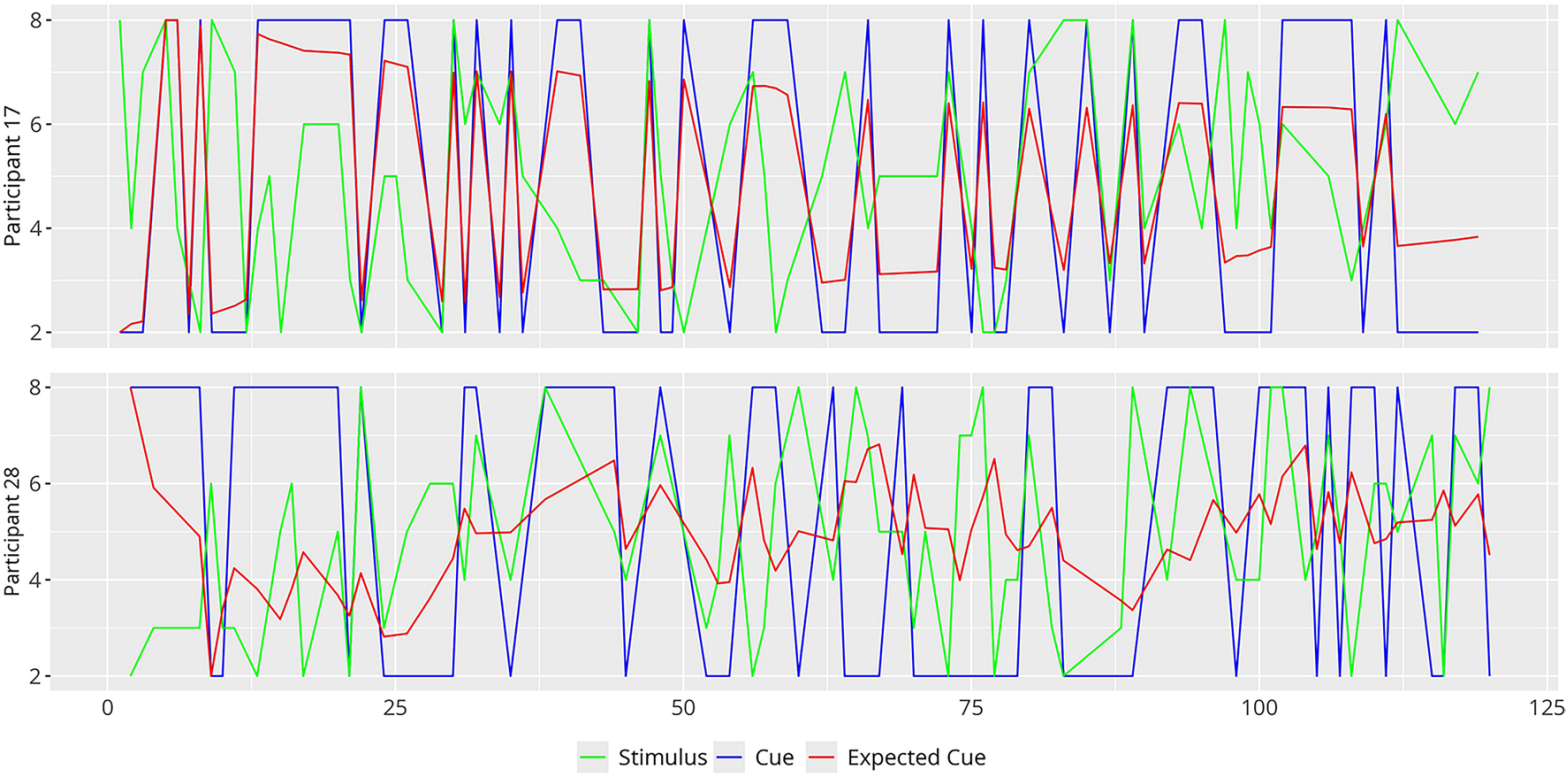
Time series plots of the Cue (green), Stimulus (blue) and Expected Cue (red) for participants 17 (highly cue driven, does not demonstrate much learning) and participant 28 (stimulus driven, demonstrates the highest rate of learning) across trials.

Figure 3 plots the actual stimulation and stimulation expected based on the nominal cue, for participants 17 and 28 in dataset 1. In veridical trials they match; but in deceptive trials, they do not. The plot depicts the value these participants assign to each cue based on Model 3.2. We focus on participants 17 and 28 as they represent two extremes in terms of how they process the cue. From the figure, it is clear that participant 17 shows minor updates in the expected cues and this remains consistent across all trials, emphasizing that this participant’s responses are primarily driven by the cue. On the contrary, participant 28’s expected cues appear to be different from the nominal cues, particularly when the prediction error is large, indicating less trust in the cue.

**Fig. 4:**
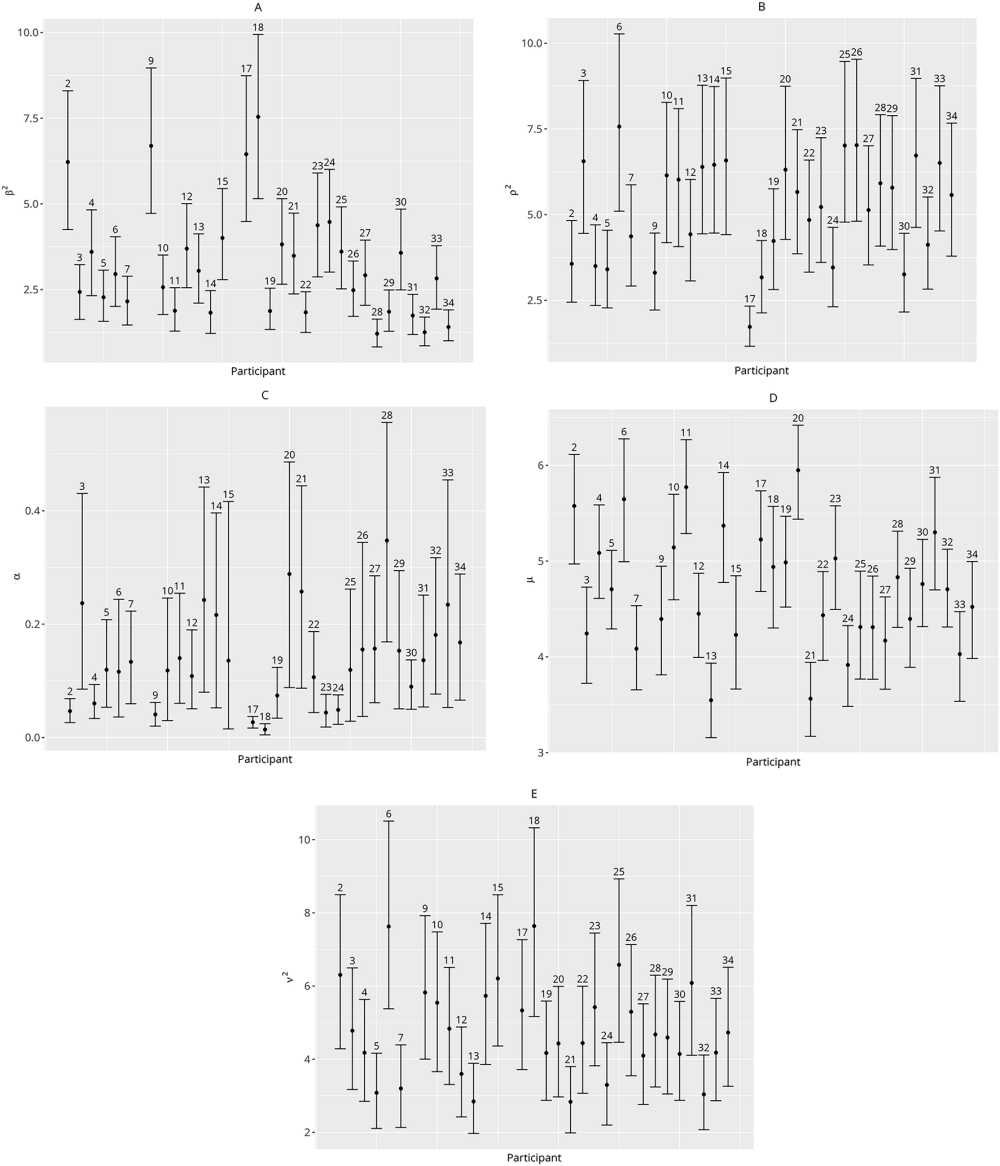
Individual level estimates with Credible Intervals (CIs) for (**A**) $\:{\varvec{\beta\:}}_{\text{i}}^{2}$ (the influence of the stimulus on participants’ pain rating), (**B**)$\:\:{\varvec{\rho\:}}_{\text{i}}^{2}$ (the influence of the expected cue on participants’ pain rating), (**C**) $\:{\varvec{\alpha\:}}_{\text{i}}$ (participants’ individual learning rate; the degree to which participants updated their beliefs based on the trial by-trial prediction error), (**D**) $\:{\:\varvec{\mu\:}}_{\text{i}}$ and (**E**) $\:{\varvec{\nu\:}}_{\text{i}}^{2}$ (the participants’ expectation for pain and the influence of their prior beliefs about pain, respectively). For each participant, each circle corresponds to the parameter estimate (i.e. posterior mean) and the line around each circle corresponds to the 95% Bayesian Credible Interval for that particular parameter, indicating a 95% probability of the parameter effect falling within the designated range of values. Shorter CIs indicate less uncertainty in the distribution of possible values for a parameter. These results correspond to Dataset 1.

To illustrate these interesting individual differences in Model 3.2, we corroborate our findings from Fig. 3 via the results in Figs. 4 and S3 in the Supplementary material. Participant 17 appears to be highly cue driven and does not show much evidence of learning ($\:{\widehat{\alpha\:}}_{17}$ and $\:{\widehat{\rho\:}}_{17}^{2}$ are small, whilst $\:{\widehat{\beta\:}}_{17}^{2}$ is large in Fig. 4; also see Figure S3 where large $\:PE$ is associated with large $\:P{E}_{subj}$). On the other hand, participant 28 is more driven by the stimulus and demonstrates the highest rate of learning ($\:{\widehat{\alpha\:}}_{28}$ and $\:{\widehat{\rho\:}}_{28}^{2}$ are large, whilst $\:{\widehat{\beta\:}}_{28}^{2}$ is small in Fig. 4; again this is supported by Figure S3, where the relevant scatterplot of $\:PE$ vs. $\:P{E}_{subj}$ appears very flat, indicating less effect of the cue). Similar conclusions can be drawn for other participants in dataset 1 as well as dataset 2; the reader is referred to Figures S2-12 in the Supplementary Materials for more information.

### Discussion

Perceived pain intensity was best explained by a model in which the expected value of the cue was updated via a Bayesian reinforcement learning algorithm. This result indicates that the subjective value of the cue changed for participants on a trial-by-trial basis, despite its fixed nominal value and the instructions that the cue was always truthful. It implies that people update their expectations about cues based on the objective stimulations they receive, even when they are told that cues are veridical – a result with repercussion for many pain perception studies.

From an adaptive perspective, it is reasonable to discard beliefs that clash with reality, because a more realistic response to pain lessens both the potential tissue damage resulting from underreacting to a painful stimulus and the potential energy wasted from overreacting to a non-painful stimulus. In our previous investigation of this dataset our modelling results agreed with this common-sense intuition, since they supported the suggestion that the effect of cues decreased when they signalled a stimulus intensity that was highly discrepant to the true stimulus intensity. However, these previous results were obtained under the assumption that participants always believed they will receive the stimulus indicated through the nominal value of the cue. This is a generally well-accepted assumption in pain and placebo research where deception using instructed cues is used^9–11,13,23,27,30^.

The current results are important because they indicate that the value of the cue changed for participants when they experienced stimulation that was discrepant to the stimulation they were instructed to expect. In a particularly clear-cut example of the issue, Tsai and colleagues have carefully examined (and rejected) potential habituation and sensitisation to the pain stimulus across their experimental session - but did not examine potential changes to the perceived value of their deceptive cues^31^. These authors found evidence that pain perception in some participants, and therefore also across the group as a whole, was best explained by a model that – like our original work – appeared to challenge established predictive coding accounts, especially those of Grahl and colleagues^14^. Our current findings suggest that it is possible that some of the participants in Tsai and colleagues’ study may have learned from repeated stimulations that cues have other than their stated nominal values, and that accounting for within-session learning could lead to different conclusions. Hence, deception studies should not assume that participants believe every cue, but instead, assume that participants update cue values over time. This finding is important for functional imaging experiments that rely on multiple repetitions of the same cued painful event to achieve higher signal-to-noise ratio, because it suggests that the expected value of cues changes rather than remaining static, as is often assumed. Our finding may also be relevant to placebo analgesia research, because it suggests that participants update their expectations about the effectiveness of a placebo treatment over time in light of sensory evidence, with implications for how well the placebo response is maintained over time. Our findings may also have clinical implications, because there is currently no appreciation that the expectations associated with open label placebo treatment might be dynamic, and this is likely to alter how the treatment influences subjective outcomes over time. This finding may only apply in situations where deception is more extreme, as in our study, where prediction error is large; with smaller errors, it is possible that the value of beliefs remains fixed. Future research could explore further the conditions which lead to fixed versus dynamic beliefs.

The Bayesian reinforcement learning model explained the data better than the Bayesian model of a trial-by-trial decrease in the influence of cues, indicating that participants were learning about the cue values, rather than dismissing the cue as time progressed. In other words, participants learned trial-by-trial, updating cue values based on the prediction error, and this learning was reflected in their pain ratings. Bayesian inference, in which prior predictions are integrated with incoming nociceptive input, provides an account for why pain perception is influenced by expectations^3–7,16^. Bayesian reinforcement learning has been proposed as the mechanism associated with pain perception^2,21^ and prediction error to pain has been found in the periaqueductal grey^32^. The dynamics of this process are still being discovered, with a recent study that used Bayesian modelling showing that participants updated their beliefs about the temporal statistics of pain trial-by-trial^33^. Our results provide support for this framework. The individual learning rate, which reflects the degree to which participants updated their beliefs based on the trial by-trial prediction error, was below 0.33 across participants and was on average 0.14, implying a modest change to the expected value of the cue as a function of learning from the stimulus. This result agrees with the reinforcement learning literature on reward-learning^34^.

Our results extend recent work modelling individual cognitive styles of pain perception^26,33,35^and we revealed additional dimensions of individual differences, such as in how much pain ratings are influenced by cues, by optimism about the stimulus intensity, and by how much prediction error they tolerated on the task before the influence of cues on pain ratings decreased. The usefulness of considering these individual differences in predicting pain ratings replicates our previous results^24,26^.

There are some limitations to this study. Our sample was young, as we recruited primarily university students, and our findings may not directly generalise to other age groups, given age-related differences in pain perception^36^. We used deception and experimental pain, so the results may be less generalisable to a naturalistic or clinical environment, although new results from our laboratory suggest that the particular modality we utilised – electric stimulation – will generalise well to other pain modalities^37^. Further, our results are specific to acute pain perception in healthy volunteers; the generalisability of our findings to patients with chronic pain conditions is currently unclear. Also, ethical considerations meant that we only included noxious stimulation that participants indicated they were willing to tolerate if administered repeatedly, whereas the effect of cues on more intense pain may differ. It would be interesting to assess whether these results hold within a clinical environment, such as in patients with chronic pain, as the results could have implications for how beliefs about pain treatments are updated over time, in line with recent work showing that expectation violations are associated with an increased ability to cope with pain, at least in a subclinical population^38^.

### Conclusions

To summarise, our key finding was that pain ratings were best explained by a Bayesian reinforcement learning model in which the value of the pain intensity cues changed for participants over the course of the experiment, indicating that the value of cues changed for participants despite its fixed nominal value and the instructions. This finding is important for theory development because it supports predictive processing models of pain perception and of perception more broadly. This finding also has practical and translational implications. It indicates that future studies assessing the influence of cues on pain perception should assume a dynamically changing cue value rather than assuming a fixed value. The finding also hints at how placebo analgesia may evolve over time and warrants further study in clinical populations and real-world settings.

## Supplementary Information

Below is the link to the electronic supplementary material.

Supplementary Material 1

## Data Availability

All data and analysis code are available at https://osf.io/fj27k/.
