# Supplementary material for: Dynamic learning of the meaning of information changes pain perception
Source: Sci Rep. 2025 Oct 14;15:35786. doi: 10.1038/s41598-025-14299-z (PMC12521376; doi:10.1038/s41598-025-14299-z)
Supplement: Supplementary file 1 — Supplementary Material 1 [file 41598_2025_14299_MOESM1_ESM.docx]

Supplementary Materials to the paper

“Dynamic learning of the meaning of information changes pain perception”

**S1. Modelling:**

In model 1, we consider the following forms for $g$:

1. $g(|PE|) = |PE|$. This implies that the variance increases as the distance between $X$ and $Z$ increases. However, this also implies the change happens linearly (as shown in Figure (a) below), which might not be a realistic assumption.
2. $g(|PE|) = 0.2exp|PE/2|$. This implies a faster rate of increase in the variance as the distance between$X$and $Z$increases (as seen in Figure (b)), which may be a more realistic assumption and in line with our findings in ^23^ that after a tipping point, the effect of the cue reduces. The 0.2 multiplier and the 0.5 scaling factor within the exponential function ensure a steeper increase around $|PE|\approx3$, which corresponds roughly to where the tipping point was observed in our previous paper^23^.
3. $g(|PE|) = log |PE|$. This implies the increase in variance slows down as the distance between$X$and $Z$increases, which contradicts our previous assumption, but is included here for completeness. Setting $g(|PE|) = 0.1$ at $|PE| = 0$ and $|PE| = 1$ (to avoid $\rho_{PE}^{2} = 0$), and $g(|PE|) = log |PE|$ otherwise, we get the function in Figure (c).


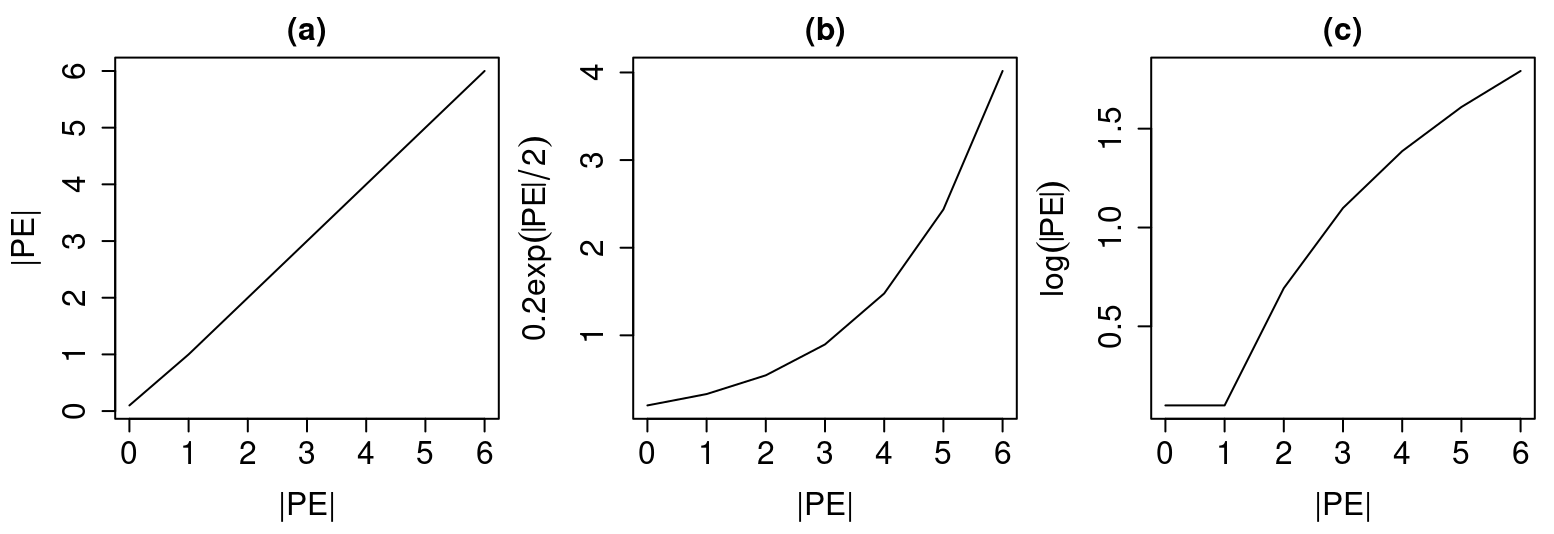


*Figure S1: Illustration of the 3 functions for* $g$*, allowing for different* *ways the variability in the cue distribution could change with the magnitude of PE.*

| *Parameter* | *Description* |
| --- | --- |
| ρ^2^ | Model 1: The additional variance in the cue not captured by the effect of the prediction error, PE. Low values indicate high influence of the cue, relative to the magnitude of PE.  Model 2: The additional variance in the cue not captured by the effect of trial. Low values indicate high influence of the cue, relative to the timing in the study.  Model 3: The variance of the cue. Indicates the weight of the expected cue on pain perception. Low values indicate high influence of the cue. |
| β^2^ | The variance of the stimulation. Indicates the weight of sensory input on pain perception. Low values correspond to high influence of stimulation. |
| μ | The mean of the trait like bias. Indicates prior expectations on pain perception, independent to the cue and stimulus. High values indicate high expected pain. |
| ν^2^ | The variance of the trait like bias. Indicates the variance of prior expectations, independent to the cue and stimulus. High values indicate a lower effect of prior expectations. |
| $\alpha$ | The learning rate. Can take values between 0 and 1. A 0 indicates that no learning has occured (participant takes the cue at face value), whereas a 1 implies no effect of the cue on pain perception. |

*Table S1: Interpretation of each parameter across our 3 models. Note that parameter* ρ^2^ *takes a different meaning in each of the 3 models.*

***S2: Results***

|  | M1.1 | M1.2 | M1.3 | M2.2 | M3.1 | M3.2 |
| --- | --- | --- | --- | --- | --- | --- |
| D1 | 28436.08 | 28000.89 | 28223.72 | 30566.51 | 27834.89 | 27655.33 |
| D2 | 27840.06 | 27567.29 | 27645.05 | 29391.59 | 27359.62 | 27384.87 |

*Table S2: Numerical DIC values for each of the models considered for both dataset 1 (D1) and dataset 2 (D2)*


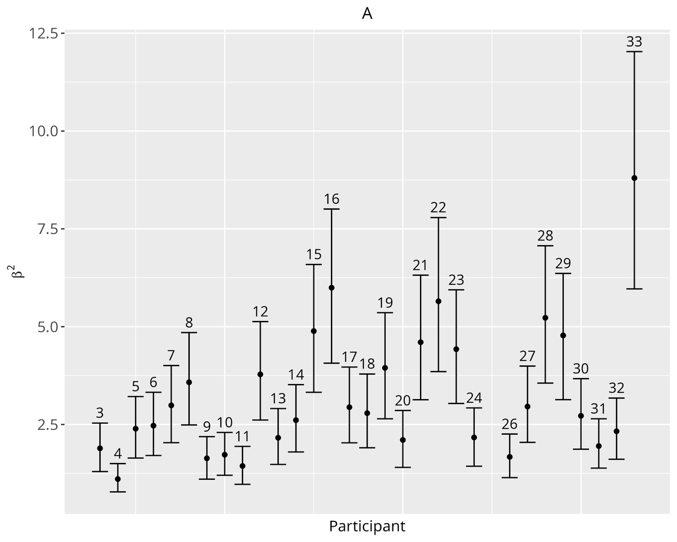

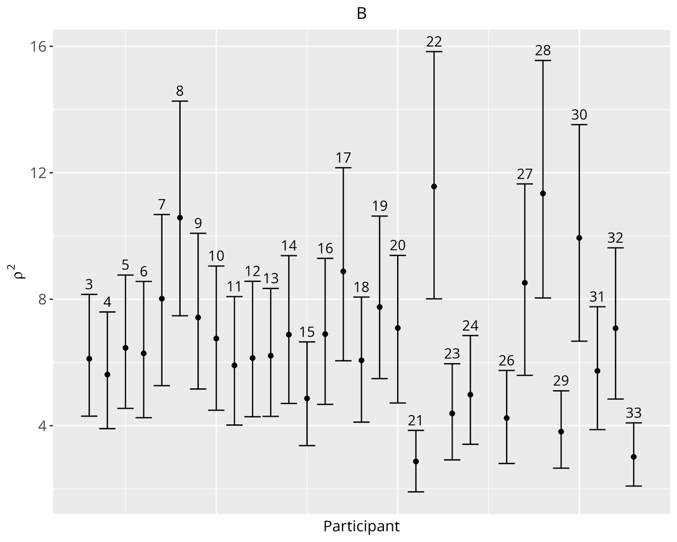

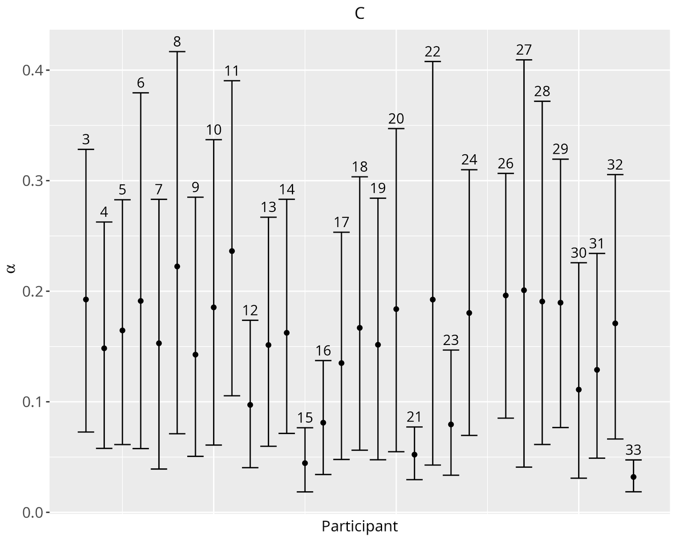

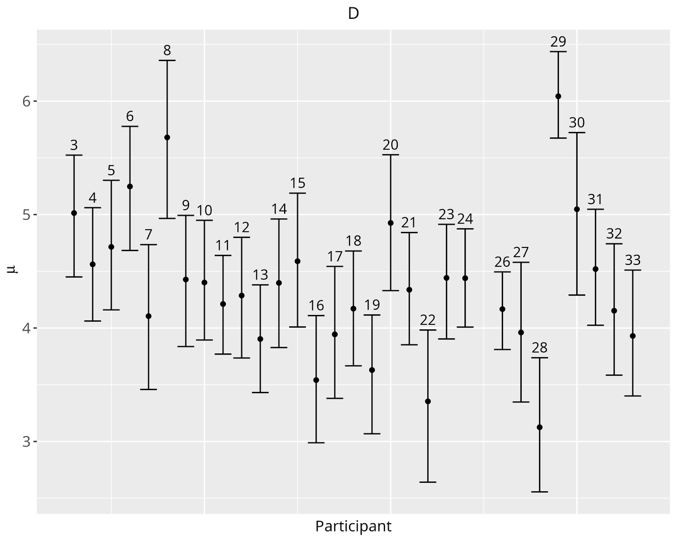

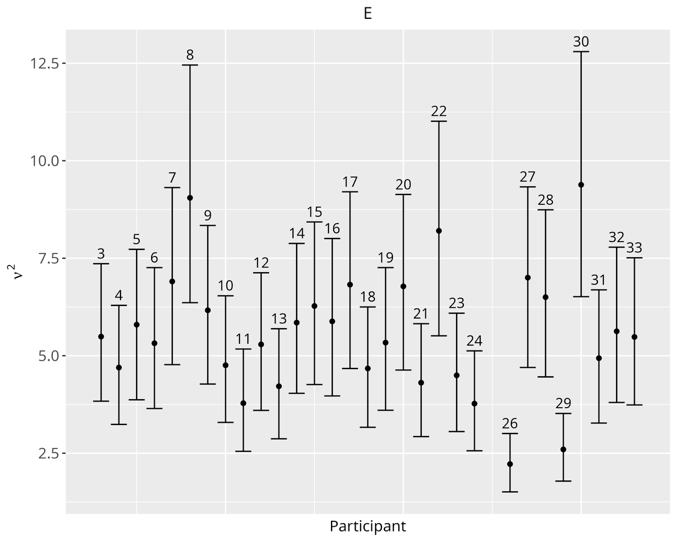


*Figure S2: Individual level estimates with Credible Intervals (CIs) for (A)* $\beta_{i}^{2}$ *(the influence of the stimulus on participants’ pain rating), (B)*$\rho_{i}^{2}$ *(the influence of the expected cue on participants’ pain rating), (C)* $\alpha_{i}$ *(participants’ individual learning rate; the degree to which participants updated their beliefs based on the trial by-trial prediction error), (D)* $\mu_{i}$ *and (E)* $\nu_{i}^{2}$ *(the participants’ expectation for pain and the influence of their prior beliefs about pain, respectively)*$.$*For each participant, each circle corresponds to the parameter estimate (i.e. posterior mean) and the line around each circle corresponds to the 95% Bayesian Credible Interval for that particular parameter, indicating a 95% probability of the parameter effect falling within the designated range of values. Shorter CIs indicate less uncertainty in the distribution of possible values for a parameter. These results correspond to Dataset 2.*


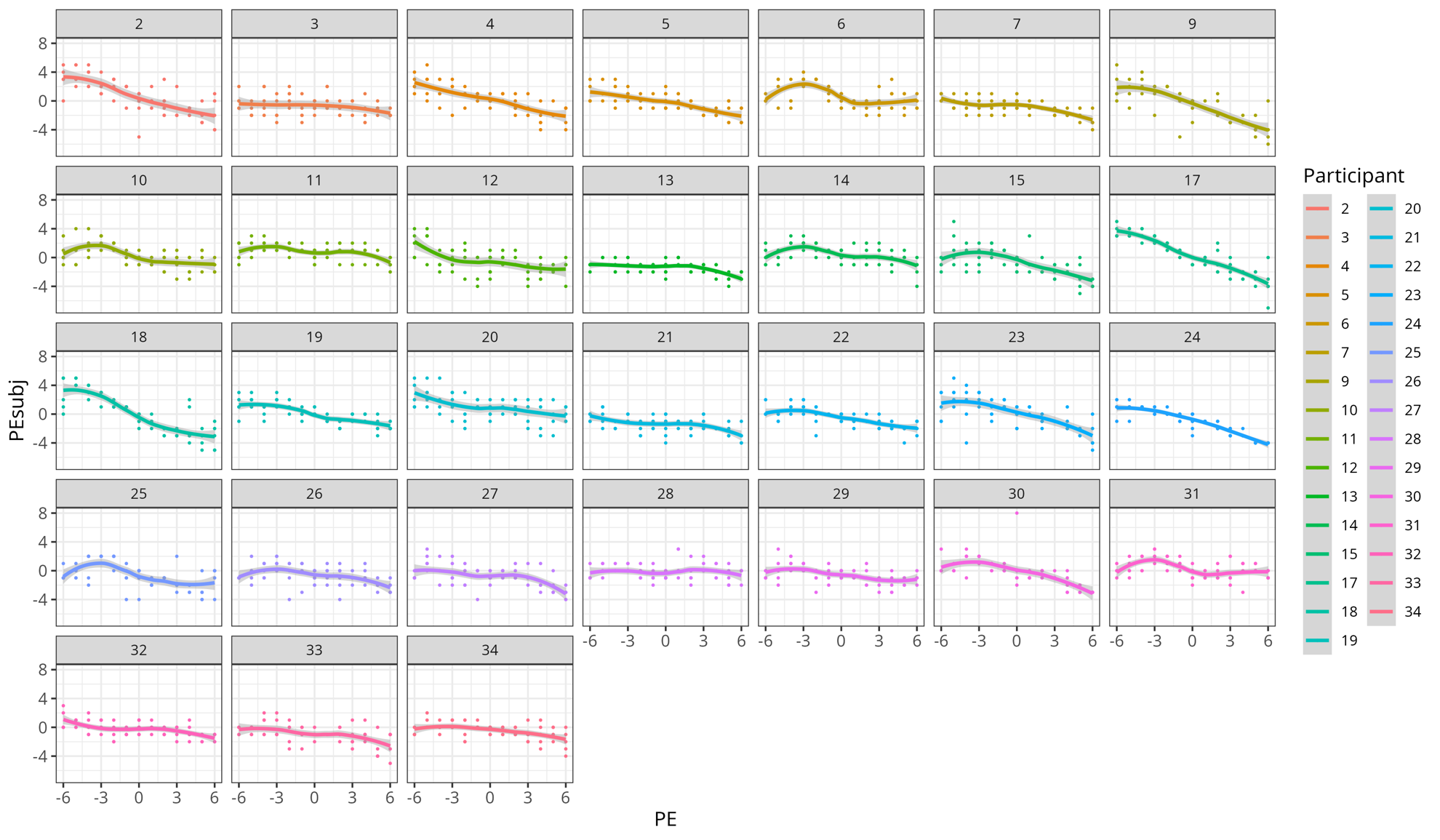


*Figure S3: Scatterplots with smooth trajectories illustrating the relationship between* $\mathrm{PE}$ *vs* $PE_{\mathrm{subj}}$ *for each participant in Dataset 1.*


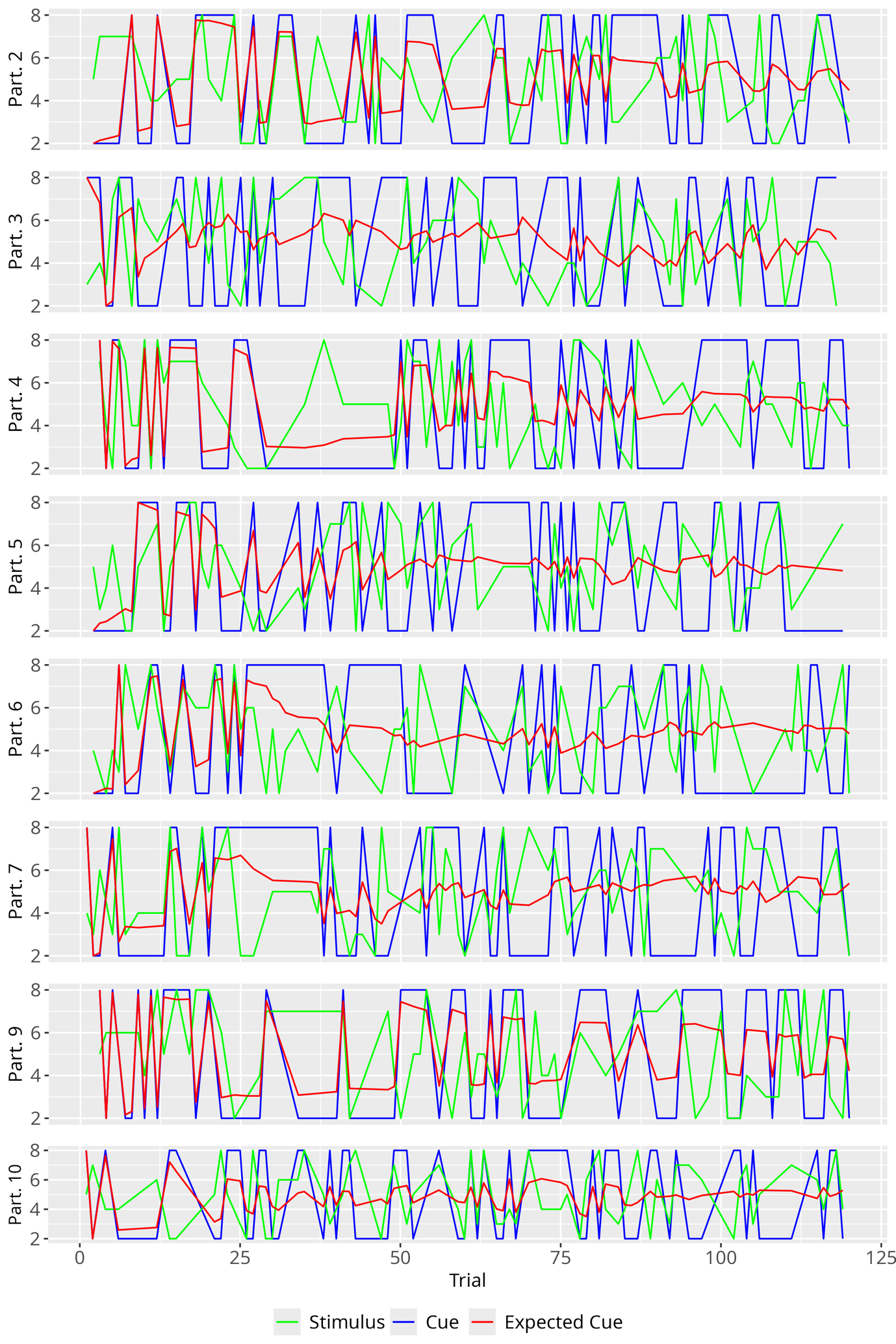
*Figure S4*
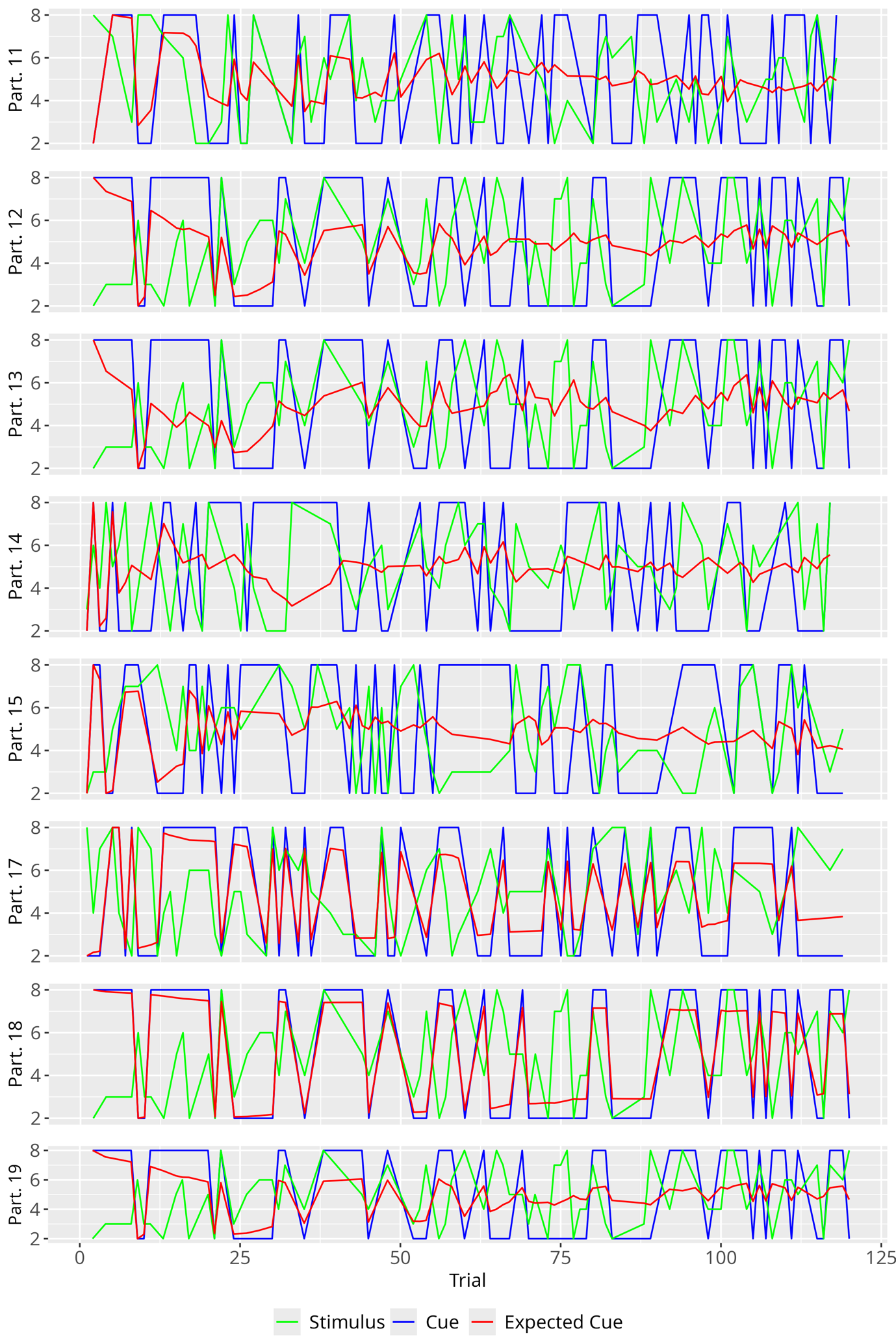
*Figure S5*
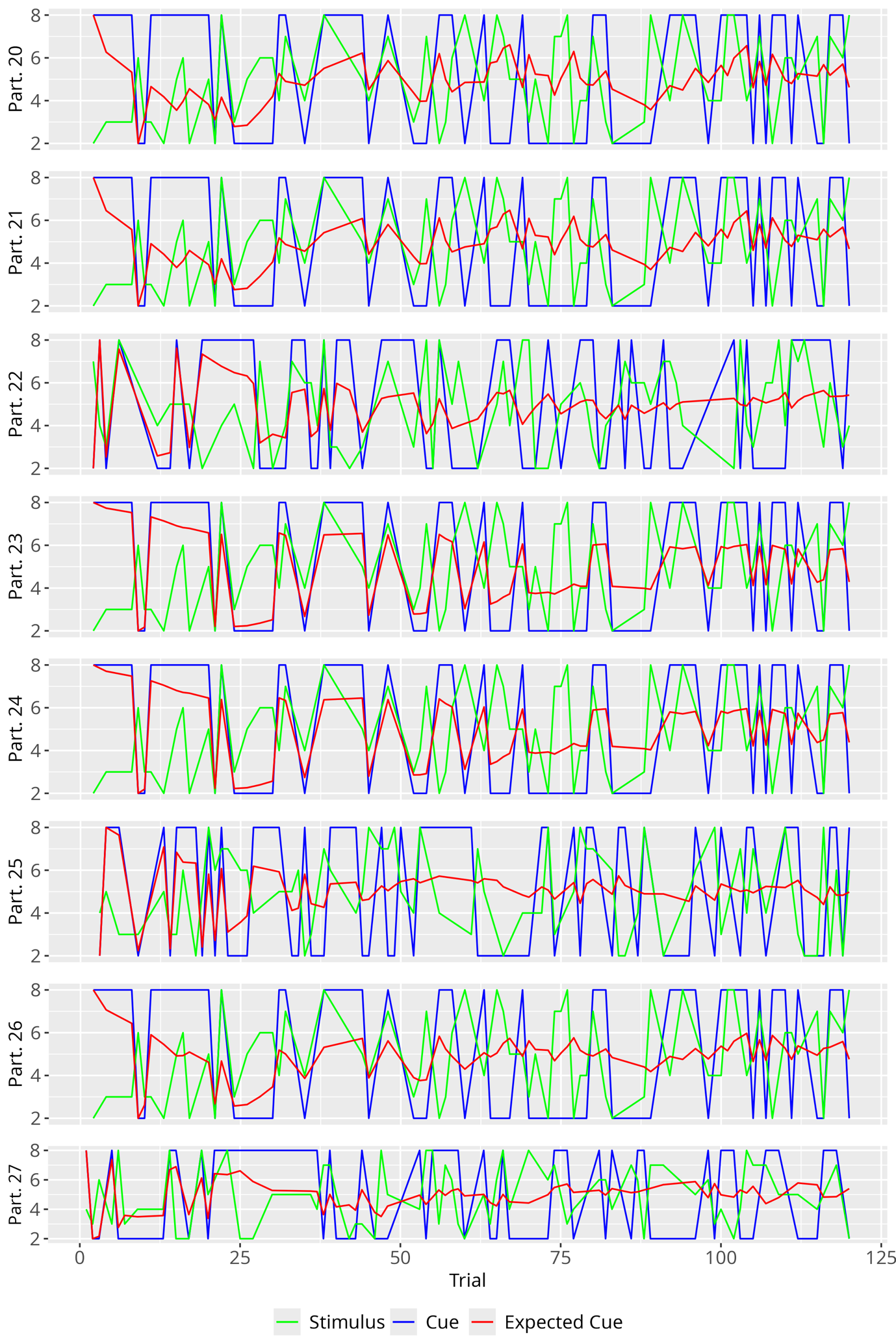
*Figure S6*
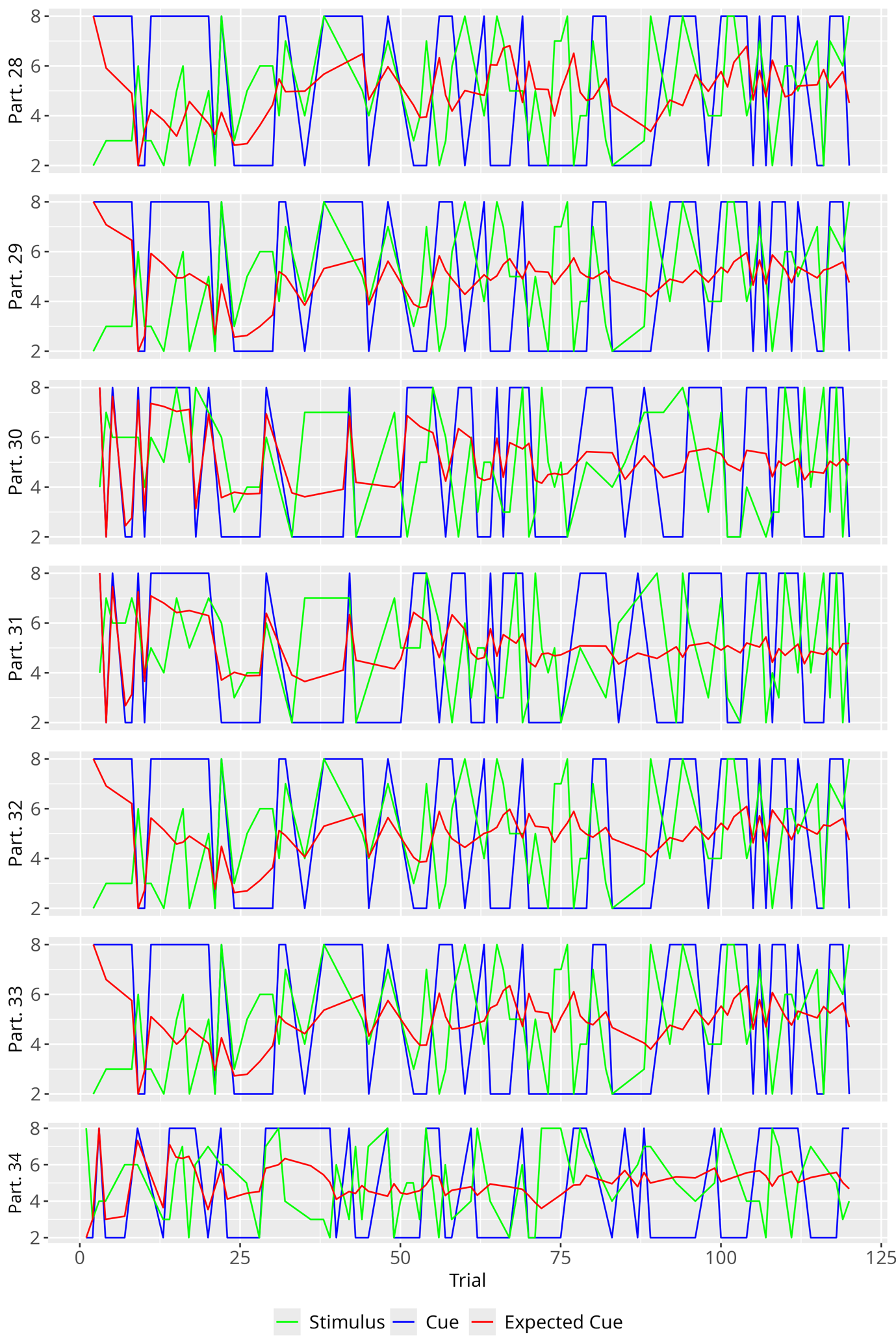


*Figure S7*

*Figures S4-S7: Time series plots of the Cue (blue), Stimulus (green) and Expected Cue (red) across trials for all participants in Dataset 1.*


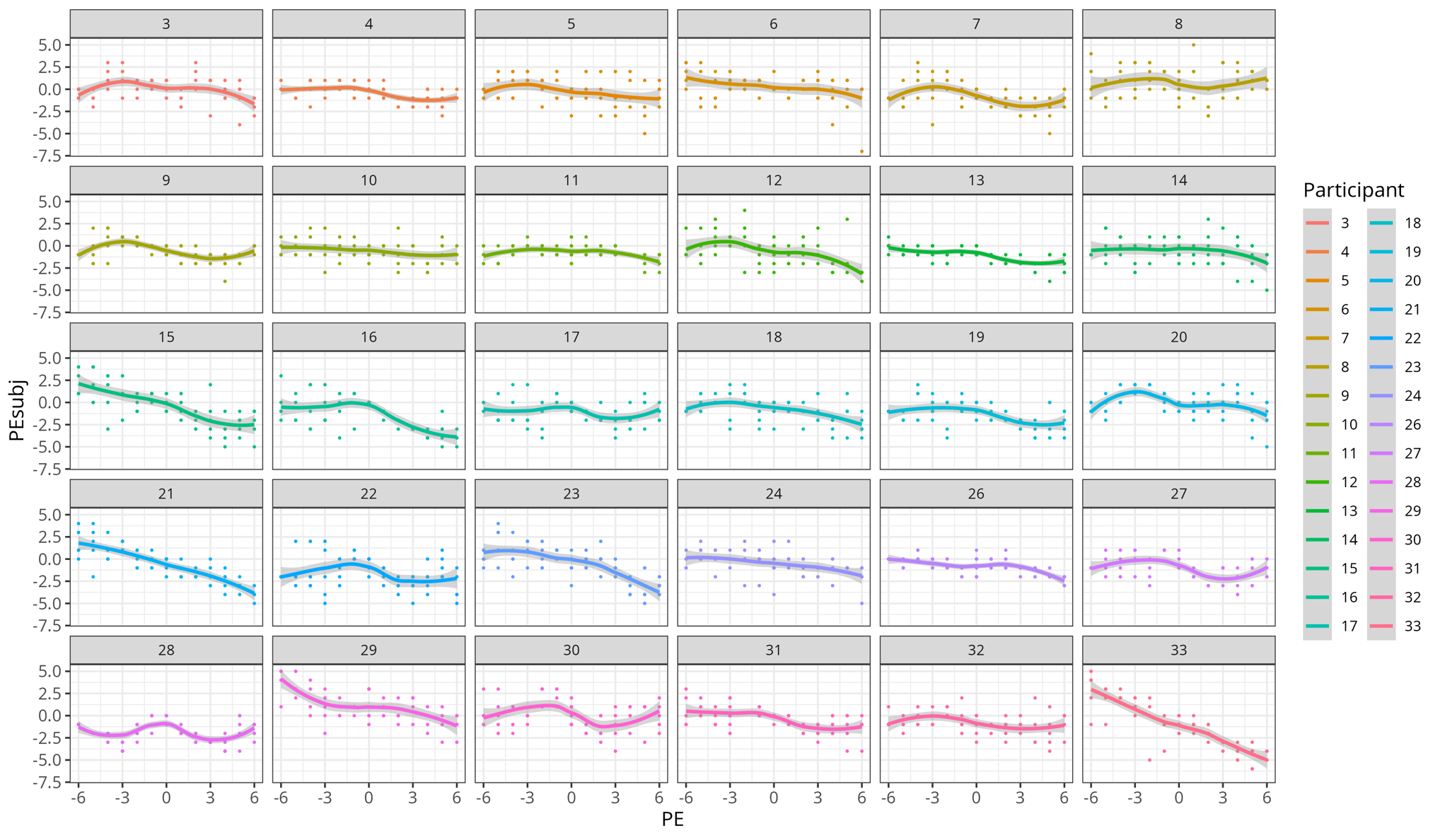


*Figure S8: Scatterplots with smooth trajectories illustrating the relationship between* $\mathrm{PE}$ *vs* $PE_{\mathrm{subj}}$ *for each participant in Dataset 2.*


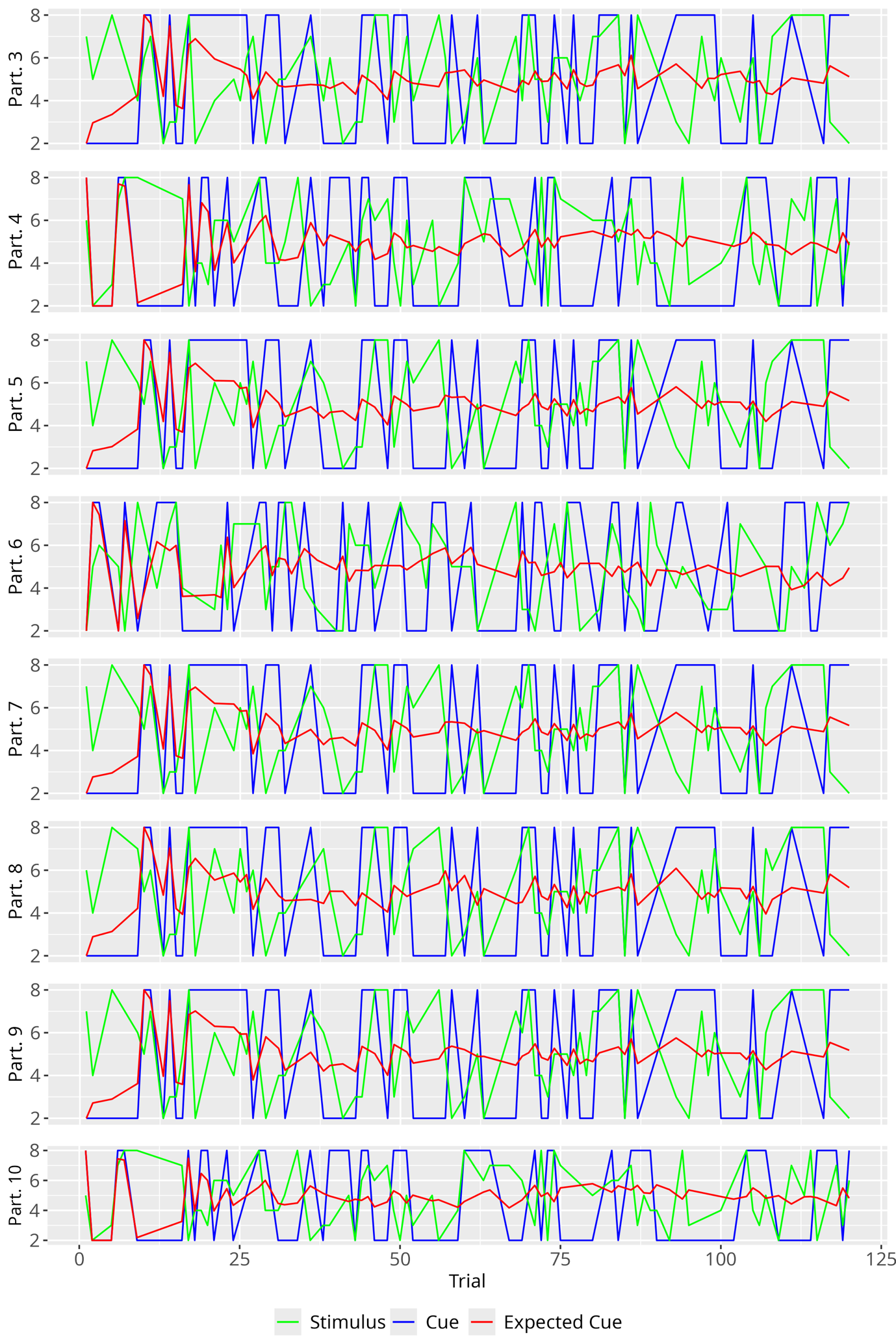
*Figure S9*


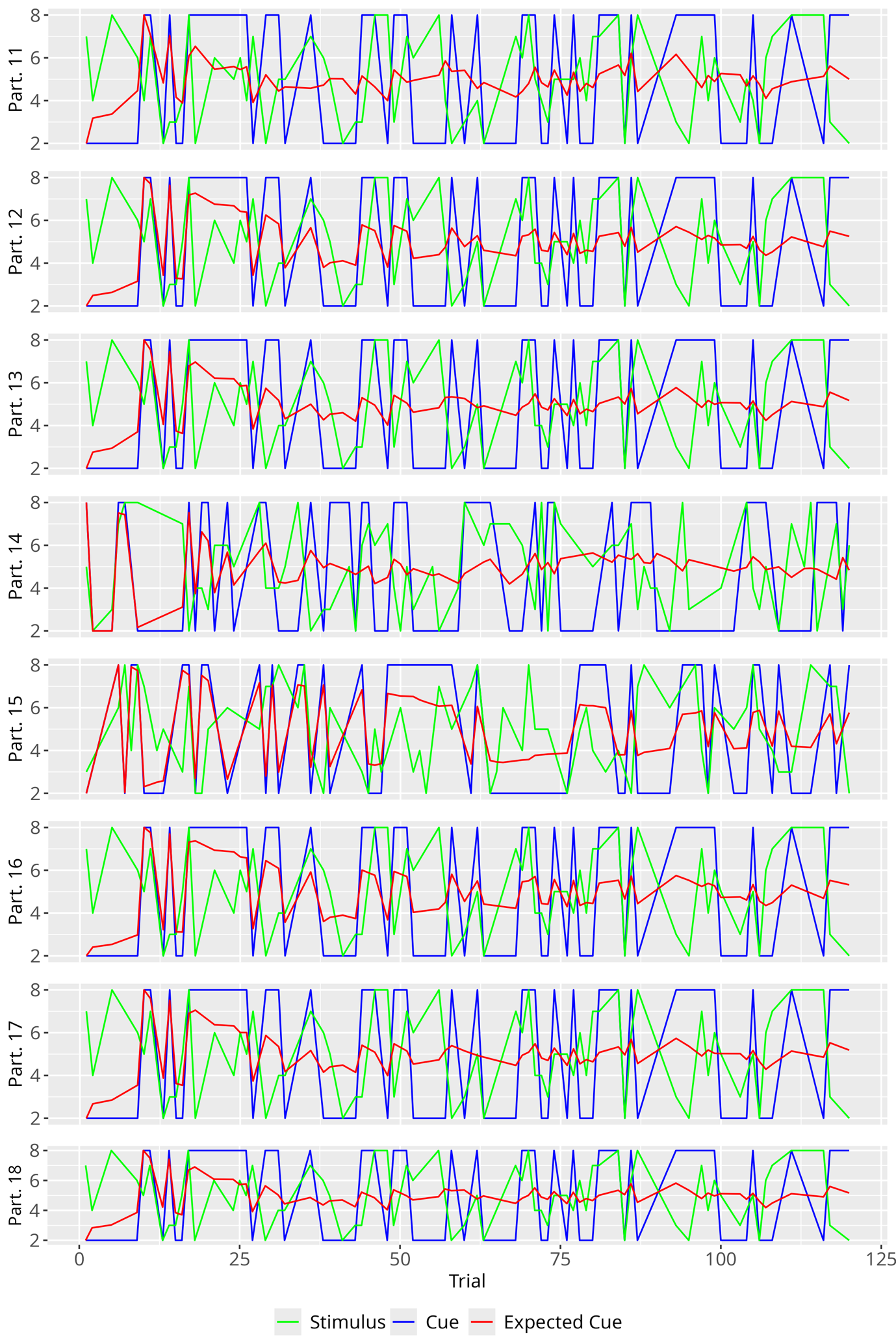
*Figure S10*


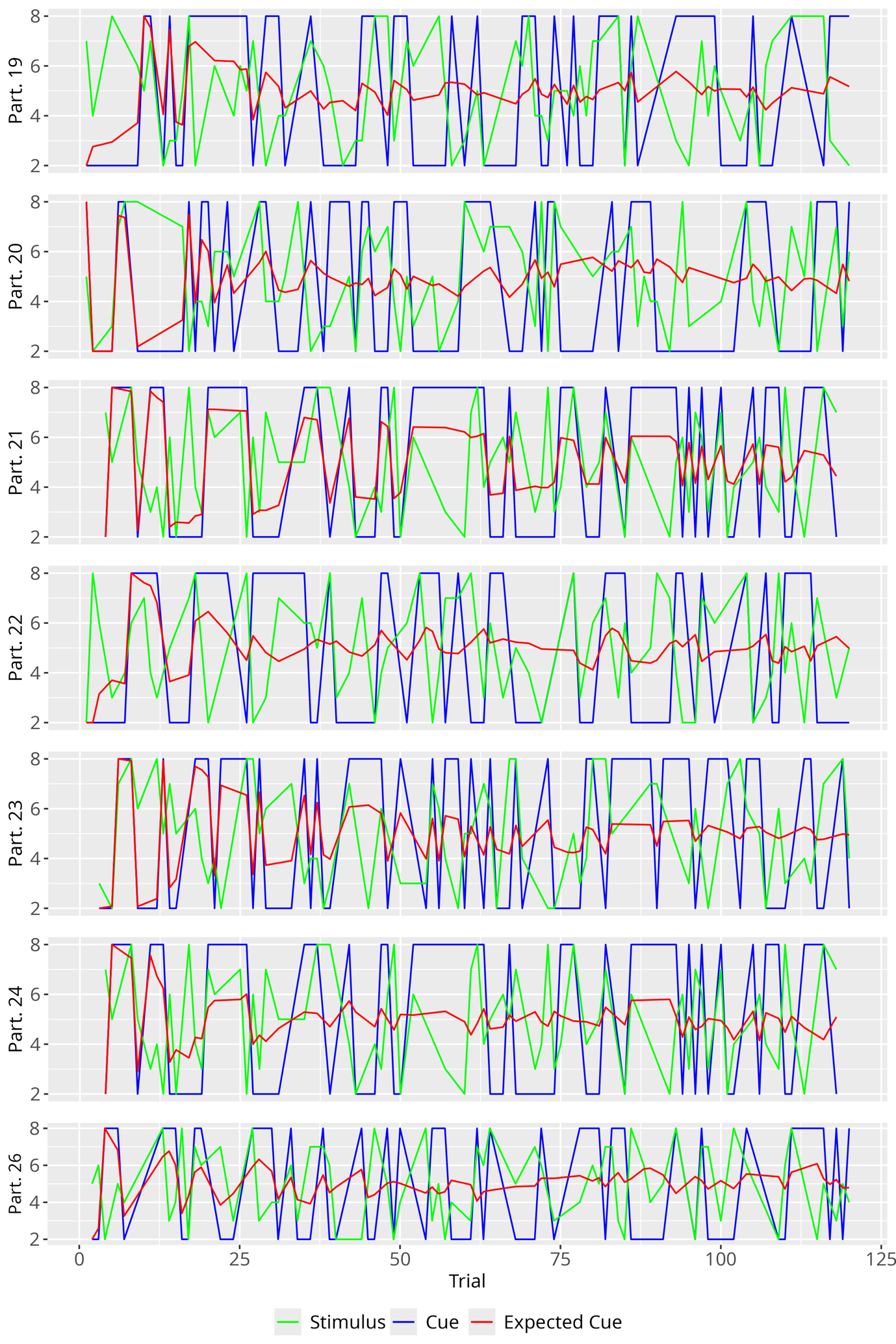
*Figure S11*


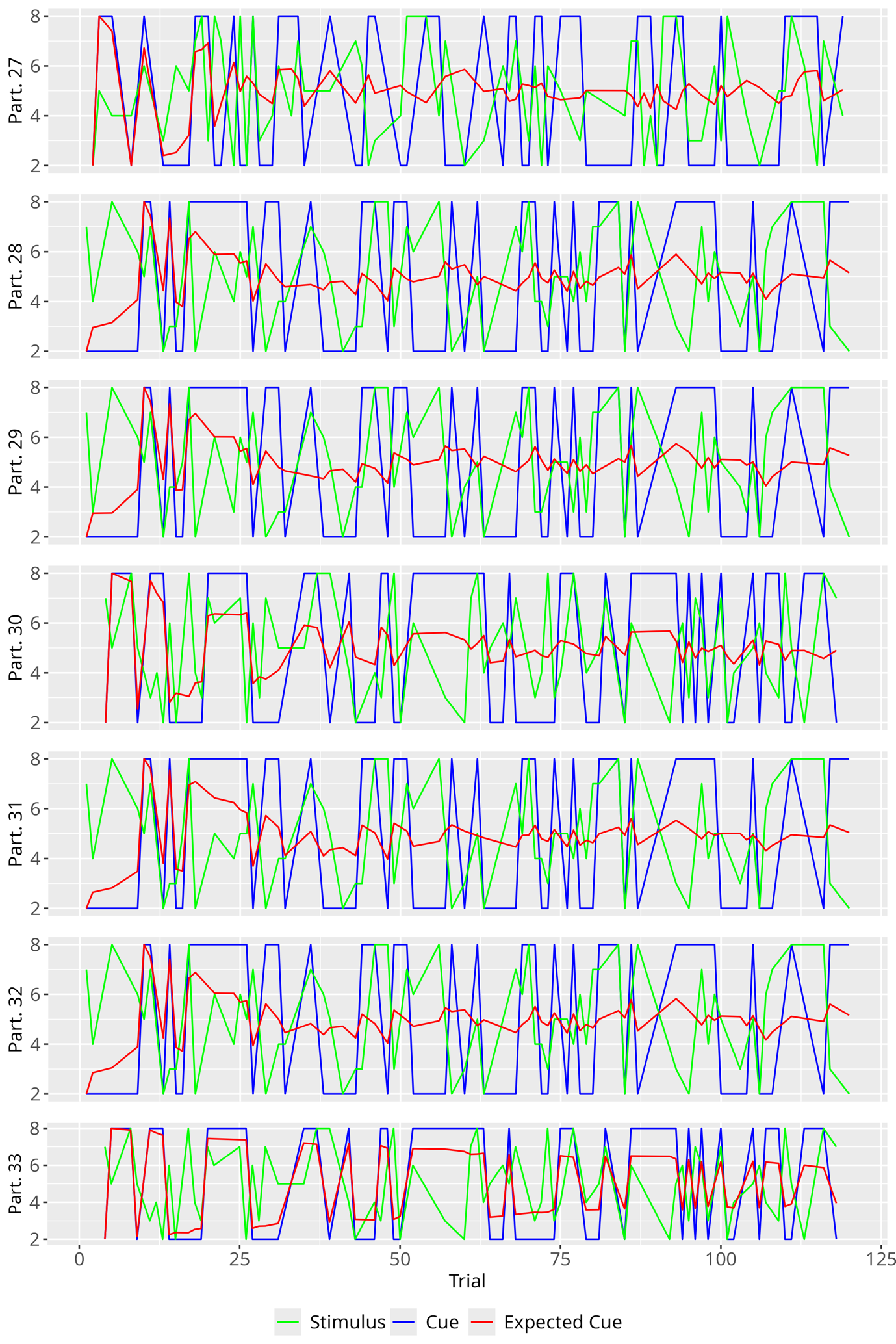
*Figure S12*

*Figures S9-S12: Time series plots of the Cue (blue), Stimulus (green) and Expected Cue (red) across trials for all participants in Dataset 2.*
